# An intradural cervical chordoma mimicking schwannoma

**Published:** 2012-11

**Authors:** Mohammad Samadian, Misagh Shafizad

**Affiliations:** ^a^Neurosurgery Department, Loghman Hakim Hospital, Shaheed Beheshti University of Medical Sciences, Tehran, Iran.

## Abstract

Chordoma is a relatively rare tumor originating from the embryonic remnants of the notochord. This is an aggressive, slow growing and invasive tumor. It occurs mostly at the two ends of neuroaxis which is more frequent in the sacrococcygeal region. Chordoma in vertebral column is very rare. This tumor is extradural in origin and compresses neural tissues and makes the patient symptomatic. This tumor found extremely rare in the spinal region as an intradural tumor.

The present study reports a rare case of intradural chordoma tumor as well as its clinical manifestations and treatment options.

**Case:**

The patient was a 50-year-old female presented with 9 months history of progressively worsening neck pain, cervical spine chordoma resembling neurinoma and right arm numbness. Physical examination showed no weakness in her limbs, but she had upward plantar reflex and mild hyperreflexia. In a magnetic resonance imaging (MRI) scan of the cervical spine there was an ill-defined enhancing mass in the posterior aspect of C2-C3 body caused cord compression more severe in right side as well as foraminal scalloping. The patient underwent surgery and after midline posterior cervical incision and paravertebral muscle stripping a laminectomy was performed from C1 through C4 using a high speed drill. Needle biopsy revealed chordoma on frozen section and all of accessible parts of tumor were excised. The gross and microscopic histopathological appearance was consistent with chordoma.

Chordomas are malignant tumors that arise from remains of embryonic notochord. These ectopic rests of notochord termed “ecchordosis physaliphora “can be found in approximately 2% of autopsies. These are aggressive, slow growing, locally invasive and destructive tumors those occur in the midline of neuroaxis. They generally thought to account for 2% to 4% of all primary bone neoplasms and 1% to 4% malignant bone neoplasms. They are the most frequent primary malignant spinal tumors after plasmacytomas. The incidence has been estimated to be 0.51 cases per million. The most common location is sacrococcygeal region followed by the clivus. These two locations account for approximately 90% of chordomas. Of the tumors that do not arise in the sacrum or clivus, half occur in the cervical region, with the remainder found in the lumbar or thoracic region, in descending order of frequency. Cervical spine chordomas account for 6% of all cases. Distal metastasis most often occurs in young patients, those with sacrococcygeal or vertebral tumors, and those with atypical histological features. These tumors usually spread to contiguous anatomical structures, but they may be found in distant sites (skin, musculoskeletal system, brain, and other internal organs). Seeding of the tumor has also been reported, and the likely mechanism seems to be tumor cell of contamination during the surgical procedures. The usual radiological findings in chordomas of spine are destructive or lytic lesions with occasional sclerotic changes. They tend to lie anterolateral, rather than dorsal towards the cord, and reportedly known to invade the dura. The midline location, destructive nature, soft tissue mass formation and calcification are the radiological hallmarks of chordomas. Computed Tomography (CT) scan is the best imaging modality to delineate areas of osteolytic, osteosclerotic, or mixed areas of bone destruction.Chordoma is usually known as a hypovascular tumor which grows in a lobulated manner. Septal enhancement which reflects a lobulated growth pattern is seen in both CT and MRI and even in gross examination. Other epidural tumors include neurinoma, neurofibroma, meningioma, neuroblastoma, hemangioma, lymphoma and metastases. Their differentiation from chordoma may be difficult due to the same enhancement pattern on CT and MRI.

A dumbbell-shaped chordoma is a rare pathogenic condition. The dumbbell shape is a characteristic finding of neurinomas in spine but in spinal neurinomas extention to transverse foramina has not yet been reported. Although our case mimicked a dumbbell shaped neurogenic tumor, its midline location and destructive pattern were characteristic feature indicating a clue to the diagnosis of chordoma that was already confirmed with histopathology.

This unusual behavior of tumor extension can be explained by the soft and gelatinous nature of the tumor enabling the mass to extend or creep into the existing adjacent anatomical structures.

**Keywords:**

Cervical Chordoma, Intradural, Computed tomography


Volume 4, Suppl. 1 Nov 2012
Publisher: Kermanshah University of Medical Sciences
URL: http://www.jivresearch.org
Copyright: (cc) BY

**Paper No. 83**

### An intradural cervical chordoma mimicking schwannoma

Mohammad Samadian [a], Misagh Shafizad [a,*]

[a] Neurosurgery Department, Loghman Hakim Hospital, Shaheed Beheshti University of Medical Sciences, Tehran, Iran.

### Abstract:

**Background:** Chordoma is a relatively rare tumor originating from the embryonic remnants of the notochord. This is an aggressive, slow growing and invasive tumor. It occurs mostly at the two ends of neuroaxis which is more frequent in the sacrococcygeal region. Chordoma in vertebral column is very rare. This tumor is extradural in origin and compresses neural tissues and makes the patient symptomatic. This tumor found extremely rare in the spinal region as an intradural tumor.

The present study reports a rare case of intradural chordoma tumor as well as its clinical manifestations and treatment options.

**Case:** The patient was a 50-year-old female presented with 9 months history of progressively worsening neck pain, cervical spine chordoma resembling neurinoma and right arm numbness. Physical examination showed no weakness in her limbs, but she had upward plantar reflex and mild hyperreflexia. In a magnetic resonance imaging (MRI) scan of the cervical spine there was an ill-defined enhancing mass in the posterior aspect of C2-C3 body caused cord compression more severe in right side as well as foraminal scalloping. The patient underwent surgery and after midline posterior cervical incision and paravertebral muscle stripping a laminectomy was performed from C1 through C4 using a high speed drill. Needle biopsy revealed chordoma on frozen section and all of accessible parts of tumor were excised. The gross and microscopic histopathological appearance was consistent with chordoma.

Chordomas are malignant tumors that arise from remains of embryonic notochord. These ectopic rests of notochord termed "ecchordosis physaliphora "can be found in approximately 2% of autopsies. These are aggressive, slow growing, locally invasive and destructive tumors those occur in the midline of neuroaxis. They generally thought to account for 2% to 4% of all primary bone neoplasms and 1% to 4% malignant bone neoplasms. They are the most frequent primary malignant spinal tumors after plasmacytomas. The incidence has been estimated to be 0.51 cases per million. The most common location is sacrococcygeal region followed by the clivus. These two locations account for approximately 90% of chordomas. Of the tumors that do not arise in the sacrum or clivus, half occur in the cervical region, with the remainder found in the lumbar or thoracic region, in descending order of frequency. Cervical spine chordomas account for 6% of all cases. Distal metastasis most often occurs in young patients, those with sacrococcygeal or vertebral tumors, and those with atypical histological features. These tumors usually spread to contiguous anatomical structures, but they may be found in distant sites (skin, musculoskeletal system, brain, and other internal organs). Seeding of the tumor has also been reported, and the likely mechanism seems to be tumor cell of contamination during the surgical procedures. The usual radiological findings in chordomas of spine are destructive or lytic lesions with occasional sclerotic changes. They tend to lie anterolateral, rather than dorsal towards the cord, and reportedly known to invade the dura. The midline location, destructive nature, soft tissue mass formation and calcification are the radiological hallmarks of chordomas. Computed Tomography (CT) scan is the best imaging modality to delineate areas of osteolytic, osteosclerotic, or mixed areas of bone destruction.Chordoma is usually known as a hypovascular tumor which grows in a lobulated manner. Septal enhancement which reflects a lobulated growth pattern is seen in both CT and MRI and even in gross examination. Other epidural tumors include neurinoma, neurofibroma, meningioma, neuroblastoma, hemangioma, lymphoma and metastases. Their differentiation from chordoma may be difficult due to the same enhancement pattern on CT and MRI.

Injury&Violence

Volume 4, Suppl. 1 Nov 2012
Publisher: Kermanshah University of Medical Sciences
URL: http://www.jivresearch.org
Copyright: (cc) BY

A dumbbell-shaped chordoma is a rare pathogenic condition. The dumbbell shape is a characteristic finding of neurinomas in spine but in spinal neurinomas extention to transverse foramina has not yet been reported. Although our case mimicked a dumbbell shaped neurogenic tumor, its midline location and destructive pattern were characteristic feature indicating a clue to the diagnosis of chordoma that was already confirmed with histopathology. This unusual behavior of tumor extension can be explained by the soft and gelatinous nature of the tumor enabling the mass to extend or creep into the existing adjacent anatomical structures.

**Keywords:**

***Cervical Chordoma, Intradural, Computed tomography***

*** Corresponding Author at:**
***Misagh Shafizad:*** *Neurosurgery Department, Loghman Hakim Hospital, Shaheed Beheshti University of Medical Sciences, Tehran, Iran.*
*Tel: 09111540432, Email: mishafizad@gmail.com,* ***(Shafizad M.).***

